# The devil is in the detail: estimating species richness, density, and relative abundance of tropical island herpetofauna

**DOI:** 10.1186/s12898-015-0049-5

**Published:** 2015-06-26

**Authors:** Harikrishnan Surendran, Karthikeyan Vasudevan

**Affiliations:** Wildlife Institute of India, Chandrabani, Dehra Dun, 248001 Uttarakhand India; Laboratory for Conservation of Endangered Species, CSIR-Centre for Cellular and Molecular Biology, Pillar 162, PVNR Expressway, Hyderguda, Hyderabad, 500048 Telangana India

**Keywords:** Rainforest, Lizards, Frogs, Precision, Relative bias, Population density, Tropical islands

## Abstract

**Background:**

One of the basic premises of drawing samples from populations is that the samples are representative of the populations. However, error in sampling is poorly recognized, and it goes unnoticed especially in community ecology. By combining traditional open quadrats used for sampling forest floor herpetofauna with intensive bounded quadrats, we explore the effect of sampling error on estimates of species richness, diversity, and density in the Andaman Islands.

**Results:**

Fisher’s α measure of species diversity and second order jackknife estimate of species richness were not sensitive to number of individuals sampled. Sampling error resulted in underestimation of density in both frogs and lizards. It influenced relative abundance of individual species resulting in underestimation of abundance of small or camouflaged species; and also resulted in low precision in lizard species richness estimates.

**Conclusions:**

Sampling error resulted in underestimation of abundance of small, fossorial or camouflaged species. Imperfect detection from less intensive sampling method results incorrect estimates of abundance of herpetofauna. Fisher’s α for species diversity and second order jackknife for species richness were robust measures. These have strong implications on inferences made from previous studies as well as sampling strategies for future studies. It is essential that these shortfalls are accounted for while communities are sampled or when datasets are compared.

**Electronic supplementary material:**

The online version of this article (doi:10.1186/s12898-015-0049-5) contains supplementary material, which is available to authorized users.

## Background

Conservation biologists are constantly faced with a conflict of interests: on the one hand, they need information on population abundance, species richness etc. for implementing conservation programs; while on the other, they should be cautious on reliability of findings from published work [1]. ‘Additional surveys’ is the most often sought after option in order to fill the gaps in knowledge. Since this is often not feasible due to limited resources, robustness of inventory is a desirable attribute of any faunal survey [2]. Robustness depends on unbiased and precise measurements of parameters that describe the biological community. However, it is a well known fact that biases in sampling are common and sampling assumptions are not always met with [3].

Since most empirical studies in community ecology use some form of sampling to derive estimates of species richness, diversity, and abundance, the properties of these samples have great practical importance [4]. Particularly, when the inventory of a community is based on a non-random fraction of a population, it results in sampling error, which precludes direct comparisons of population size and species diversity measures across studies. To address this, some fundamental questions about survey methods need to be answered: how are bias and precision of estimates of diversity and abundance related to survey methods and effort? How do they affect our interpretation of estimates of population sizes and subsequent conservation decisions? Answering these questions and overcoming the problems in sampling are central to good-practice in data collection and study design.

We illustrate this problem using a small tropical island herpetofauna. Small island fauna compose of a finite community and they could be sampled using quadrat sampling with varying levels of intensity. Several studies in the tropics have used quadrats as sampling units to document patterns in species richness, distribution, and abundance of forest-floor herpetofauna [5–18]. Most of these studies have assumed that they recorded all individuals and species in the quadrats during sampling, i.e. no sampling error, which is a major assumption (but see [13, 14, 19]). We explored the effect of sampling error on species richness, diversity, and density estimates from such ‘open quadrats’ (OQ) by combining them with ‘bounded quadrats’ (BQ) that gave total counts of individuals in the sampled area [13]. Our study revealed that intensive sampling using BQ had varying effects on species richness and diversity, depending on the estimator, while it significantly improved density estimates.

## Results

### Effect of quadrat characteristics

**Figure 1:**
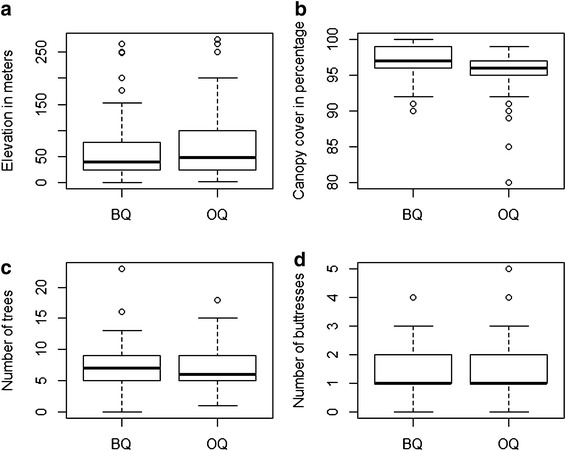
Variation in environmental variables between open and bounded quadrats **a** Elevation **b** Canopy cover **c** Number of tress **d** Number of buttresses.

Boxplots of environmental variables showed no major difference between OQ and BQ (Figure 1). Further, ANCOVA indicated quadrat type to be the most significant factor explaining the variation in number of individuals (lizards—residual standard error = 8.29 on 191 degrees of freedom (DF), multiple *R*^2^ = 0.24, adjusted *R*^2^ = 0.21, *F* = 8.65 on 7 and 191 DF, *p* = 3.31 × 10^−09^; frogs—residual standard error = 4.95 on 185 DF, multiple *R*^2^ = 0.22, adjusted *R*^2^ = 0.19, *F* = 8.65 on 7 and 185 DF, *p* = 6.02 × 10^−08^; Table 1). Canopy cover also influenced number of both lizards and frogs recorded. Number of buttressed trees and number of trees influenced abundance of lizards and frogs respectively. In general, habitat characteristics did not overwhelmingly influence abundance of frogs and lizards recorded in quadrats.

**Table 1:** Analysis of covariance table for number of individuals recorded in quadrats with number of individuals of lizards and frogs as the independent variable and quadrat type, forest type, elevation, number of trees, canopy cover and number of tree buttresses as predictor variables

	DF	SS	MS	F	Pr(>F)
Lizards					
Quadrat type	1	3,151.70	3,151.70	45.89	1.51 × 10^−10^
Forest type	2	52.90	26.46	0.39	0.68
Elevation	1	26.60	26.60	0.39	0.51
Trees	1	14.80	14.75	0.22	0.64
Canopy cover	1	375.00	374.99	5.46	0.02
Buttress	1	536.50	536.49	7.81	0.01
Residuals	191	13,118.00	68.68		
Frogs					
Quadrat type	1	869.60	869.60	35.46	1.28 × 10^−08^
Forest type	2	34.90	17.46	0.71	0.49
Elevation	1	34.40	34.36	1.40	0.24
Trees	1	163.00	163.01	6.65	0.01
Canopy cover	1	159.20	159.16	6.49	0.01
Buttress	1	26.60	26.58	1.08	0.30
Residuals	185	4,536.40	24.52		

### Species richness

**Figure 2:**
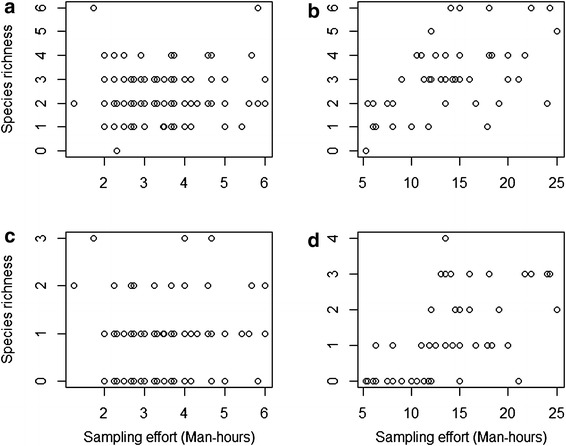
Relationship between sampling effort and species richness **a** Lizards in OQ **b** Lizards in BQ **c** Frogs in OQ **d** Frogs in BQ.

**Figure 3:**
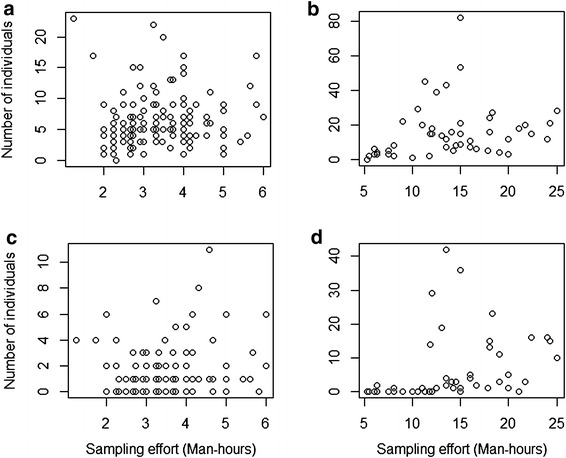
Relationship between sampling effort and abundance **a** Lizards in OQ **b** Lizards in BQ **c** Frogs in OQ **d** Frogs in BQ.

**Figure 4:**
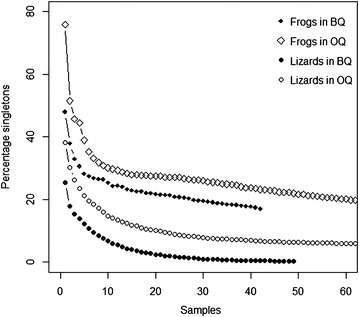
Detection of rare species in the community expressed as reduction in percentage of singletons against sample size for frogs and lizards in open and bounded quadrats. *Solid diamonds* frogs in BQ, *open diamonds* frogs in OQ, *solid circles* lizards in BQ, *open circles* lizards in OQ.

Observed species richness, of both lizards and frogs, were similar in BQ and OQ (Table 2). For both lizards and frogs, Chao1 estimators were significantly different between OQ and BQ for both lizards (*t* = 9.90, df = 46, *P* < 1.0 × 10^−5^) and frogs (*t* = 3.57, df = 41, *P* = 9.29 × 10^−4^). Fisher’s α was also different between OQ and BQ for both lizards (*t* = 5.18, df = 756, *P* < 1.0 × 10^−5^) and frogs (*t* = −4.11, df = 187, *P* = 5.8 × 10^−5^). Second order jackknife estimate was not influenced by sampling methods i.e., the way individuals were included in the samples (Table 1). Species richness of both frogs and lizards were correlated with sampling effort in BQ (Pearson’s product-moment correlation, frogs *r* = 0.58, *t* = 4.87, *P* = 1.30 × 10^−05^; lizards, *r* = 0.54, *t* = 4.38, *P* = 6.68 × 10^−05^; Figure 2b, d but not in OQ (frogs, *r* = 0.08, *t* = 0.99, *P* = 0.33; lizards, *r* = 0.04, *t* = 0.52, *P* = 0.60; Figure 2a, c). Abundances of frogs and lizards were not correlated with sampling effort in both the methods (Figure 3). Accumulation of rare species in samples (singletons) was faster for frogs and lizards in BQ than in OQ (Figure 4).

**Table 2:** Summary of analysis of species richness and density using bounded quadrats and open quadrats

Bounded quadrats							Open quadrats					
	n	S_obs_	S_Chao1_	S_Jack2_	α	D	S_obs_	S_Chao1_	S_Jack2_	α	D	
Lizards	47	7	7 ± 0.03	6.99 ± 0.09	1.07 ± 0.15	15.45 ± 15.55	7	6.32 ± 0.47	6.86 ± 1.64	1.03 ± 0.15	6.15 ± 4.09	
Frogs	42	8	7.62 ± 1.58	7.95 ± 2.5	1.25 ± 0.23	7.19 ± 10.24	7	6.19 ± 2.06	7.53 ± 2.84	1.35 ± 0.24	1.31 ± 10.24	

Though 49 bounded quadrats were sampled, for the purpose of comparison of estimated species richness, we use a sample size of 47 for lizards because beyond this, the standard deviation of the estimated species richness became zero for jackknife2 estimate. Estimated species richness of frogs is compared at a sample size of 42 as the remaining 7 bounded quadrats were in small islands where no amphibian has ever been recorded. Fisher’s α is compared at a common number of individuals (757 lizards and 188 frogs). The standard deviations for density estimates presented here are unadjusted for sample size difference.

*n* number of quadrats based on which the estimates were arrived at, *S*_*obs*_ observed species richness, *S*_*Chao1*_ Chao1 estimate, *S*_*Jack2*_ second order jackknife estimate, *α* Fisher’s alpha, *D* density (individuals/100 m^2^).

### Density and relative abundance

We detected 757 lizards and 302 frogs in 49 and 42 BQ respectively. From 151 OQ, we detected 929 lizards and 188 frogs. When the variance was adjusted for sample size difference, density estimates from BQ were significantly greater than that from OQ for both lizards and frogs (Wilcoxon–Mann–Whitney test, lizards, W = 5,209, *P* = 1.67 × 10^−5^; frogs, W = 4,165, *P* = 1.09 × 10^−4^). In the case of lizards, the density estimate from BQ was more than twice that from OQ (Table 2). In the case of frogs, density estimate from BQ was more than seven times that from OQ (Table 2). However, the effort involved in a BQ was significantly more (16.75 ± 4.02 man-hours) than that for an OQ (3.39 ± 0.97 man-hours; Wilcoxon-Mann–Whitney test, W = 4,832, *P* < 2.2 × 10^−16^).

**Figure 5:**
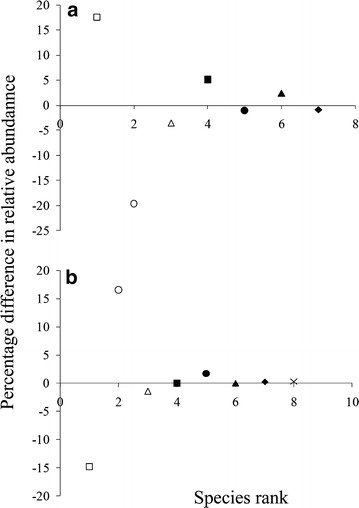
Percentage difference in relative abundance of (**a**) lizards and (**b**) frogs in open and bounded quadrats. Species ranks are those from bounded quadrats. Positive deviations show greater relative abundance in open quadrats and negative deviations show greater relative abundance in bounded quadrats. **a** Open square, *Coryphophylax subcristatus*; open circle, *Lygosoma bowringii*; open triangle *Cyrtodactylus rubidus*; solid square, *Coryphophylax brevicaudus*; solid circle, *Cnemaspis* sp.; solid triangle, *Eutropis andamanensis*; solid diamond, *Sphenomorphus maculatus*. **b** Open square, *Limnonectes* cf. *doriae*; open circle, *Ingerana charlesdarwini*; open triangle, Bush toad; solid square, *Duttaphrynus melanostictus*; solid circle, *Microhyla chakrapanii*; solid triangle, *Fejervarya cancrivora*; solid diamond, *Fejervrya* cf. *andamanensis*; multiplication sign, *Kaloula baleata ghoshi*.

There was a noticeable difference in the relative abundance of some species between the two methods. Among lizards, the small, fossorial species *Lygosoma bowringii* showed the greatest increase in relative abundance within BQ, while the larger and more conspicuous *Coryphophylax subcristatus* and *Eutropis andamanensis* had greater relative abundance in OQ (Figure 5). Among frogs, the small leaf-litter dwelling *Limnonectes* cf*. doriae* had greater relative abundance in bounded quadrats, while the arboreal and brightly coloured *Ingerana charlesdarwini* had greater relative abundance in OQ than in BQ (Figure 5).

### Relative bias and precision

Density estimates obtained from OQ had high relative bias and low relative precision for both lizards (relative bias = −0.40, relative precision = 0.51) and frogs (relative bias = −0.74, relative precision = 0.33). Second order jackknife estimate of species richness had low relative bias for frogs (−0.02) and lizards (−0.01). The relative precision of the estimate for lizards was also low (1.26) and in frogs it was high (0.06). Species diversity estimated as Fisher’s α, of frogs and lizards, the relative bias and relative precision ranged from −0.02 to 0.03, implying no difference in the efficiency of the methods for this parameter.

## Conclusions

Stemming rapid loss of biodiversity in the tropics requires robust scientific findings that bolster conservation decisions. It is up to the field biologists to ensure that the results provided are accurate so that decisions on resource utilization and management are not based on biased or imprecise results. In most community studies on herpetofauna, sampling methods are chosen arbitrarily, as a result, little attention has been paid to the effect of sampling error and its implications on inferences drawn [13, 20–23]. Recently, Kellner and Swihart [20] showed that 77% of ecological studies ignored imperfect detection, while the rest accounted for this through estimation of detection probability through statistical modelling. A majority of studies (86%) also reported detection probability to be highly variable, inflating the error in estimated parameters [20]. Our study showed that sampling error in open quadrats resulted in bias in estimated population density and in detection of less conspicuous and small frogs and lizards. Open quadrats have often been used repeatedly for sampling forest floor herpetofauna and these studies report accurate estimates of density from such plots. However, Rodda et al. [13, 19] reported high densities of reptiles using their ‘total removal plots’, which is a form of bounded quadrat. Bounded quadrats have invariably reported high densities of herpetofauna [13, 21, 24–26]. Our data revealed that bounded quadrats are efficient in sampling rare species and we expect that its performance will be better in tropical mainland areas than in islands, where a large proportion of the community is composed of rare species. This study provides empirical evidence for second order jackknife estimate for species richness and Fisher’s α for diversity as robust measures as they were not sensitive to the number of individuals included in the samples. Intensive sampling influenced measures of relative abundance of species and thereby altered community structure. Relative bias and precision point at high overall efficiency of bounded quadrats. We anticipate that our findings would prompt more field ecologists to consider careful selection of methods, analyses of data, and comparison of results from past studies. A promising direction of further research is the use of bounded quadrats for calibration of estimates obtained from other less intensive sampling methods [26]. This would pave way for developing new and more efficient sampling methods suitable for use in the tropics. At present, there is no alternative to an intensive sampling method to obtain precise or unbiased estimates of abundance and species richness of herpetofauna in tropical areas. We emphasize the need for refinement in sampling techniques so that credible findings strengthen tropical conservation.

## Methods

Forest-floor herpetofaunal community is usually sampled using litter plots or quadrats demarcated on the ground [27–29]. This method assumes that all or most individuals within the quadrat are detected without any error in sampling [13]. This assumption is often flouted, as the quadrats are not fenced (hereafter referred to as ‘open quadrats’ or OQ), and many individuals escape or remain undetected. Fenced quadrats (hereafter referred to as ‘bounded quadrats’ or BQ) prevent escape during sampling thereby capture large number of animals [13, 19, 22, 26].

### Study area

**Figure 6:**
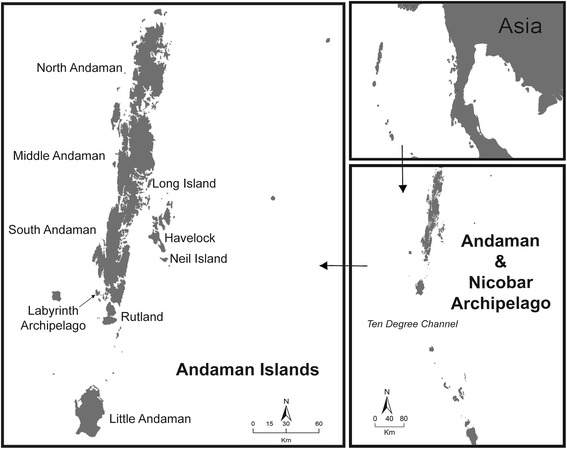
The Andaman Islands. The labyrinth Archipelago has 15 small islands, among which eight were sampled during this study. This map was made by S. Harikrishnan.

The Andaman and Nicobar Islands consist of 556 islands, islets and rocks in the Bay of Bengal (Figure 6). These islands are part of a north south running submerged mountain chain from the ArakanYoma of Myanmar to the Mentawei Islands near Sumatra, Indonesia and show distinct biogeographic affinities [30–32]. Annual rainfall in these islands exceeds 3,000 mm, and the dominant vegetation types are wet evergreen, semi evergreen and mangrove forests [33]. For a general description of these islands and their herpetofauna, see Das [31]. We sampled 15 islands in the Andaman Islands covering a large range of island areas.

### Sampling design

The evergreen forests of Andaman Islands are generally of low elevation and gentle slope [34], and in order to avoid variation in estimates due to different site characteristics, we restricted sampling to elevations between 0 and 275 m asl with mean elevation of all quadrats being 74 ± 5 m. For all quadrat sampled We recorded the following microhabitat variables: forest type (Wet evergreen or WEG, Secondary evergreen or SEG, and Littoral evergreen or LEG), elevation in meters, number of trees with gbh >20 cm, number of trees with buttress roots, and canopy cover in percentage (Additional file 1). We sampled 151 open quadrats (OQ) and 49 bounded quadrats (BQ) during the relatively dry season that lasts from November to May, between 2010–2011 and 2011–2012 (Additional file 2). No quadrats were sampled during the wet season due to logistical difficulties in conducting field work during that period in these islands (Figure 6) (Additional file 3).

We placed OQ (10 m × 10 m) randomly in evergreen forest in all islands. They were demarcated with a nylon rope and four pegs at the four corners. Four to five people searched these quadrats following standard procedure [5, 6, 27]. We sampled 151 such quadrats. We counted individuals that escaped the quadrat before capture, but these records were used only in the calculation of overall densities and not for species richness and diversity estimates because it was not possible to identify such individuals to species level without closer examination. In order to compare the bias and precision of OQ in sampling forest floor herpetofauna, we also sampled BQ of the same dimension, in the same sites where OQ were sampled. We could not place these quadrats randomly because of uneven terrain, hard or rocky substrate, or large fallen tree trunks. We placed BQs in rainforests with relatively flat terrain (all quadrats below 270 m elevation) devoid of large fallen trees and large rocks [9, 13]. However, care was taken to place them in similar habitats as OQ in order to reduce variation due to differences in habitat characteristics and within an island site, all BQ were placed within the general area covered by all the OQ in that island site. Because of the disturbance created in sampling a single quadrat, a minimum distance of 50 m was maintained between all quadrats (OQ and BQ). Our BQ differed from Rodda et al. [13, 19], in that we used a 0.5 m high plastic sheet with its bottom buried in soil. We did not grease the top of the sheet, as our study area had only two species of lizards with expanded subdigital lamellae, which were arboreal species, and were recorded rarely within quadrats. Once established, we detached the top of the plastic sheet from the supporting stakes, so that it fell flat on the ground and left it undisturbed for about 24 h. The following day, we approached the quadrat from four sides and quickly raised the plastic sheet, securing the top edge to the stakes so that no animals escaped. We sampled within the BQ following Rodda et al. [13], with the exception that we did not remove trees. Instead, we applied a broad strip of smooth duct tape around the trunk of trees at a height of 2 m from the ground, which effectively prevented all lizards other than two species with expanded sub-digital lamellae from arboreal escape. We captured all arboreal agamid lizards in the quadrats using a fishing line noose at the beginning of the quadrat search. We scanned tree trunks up to a height of 2 m and captured all frogs and lizards resting on top of or under the bark of trees.

### Data analysis

We pooled the data from all islands for estimating species richness and density. Therefore, the densities reported here are average densities across the Andaman Islands. First, we explored the differences between OQ and BQ in environmental variables through boxplots. We also explored the effect of these variables and the types of quadrats on number of individuals detected in quadrats through General Linear Models (GLM) using an Analysis of Covariance (ANCOVA). In this analysis, number of individuals detected was used as response variable and quadrat type (OQ and BQ), forest type (WEG, SEG and LEG), elevation, number of trees, number of trees with buttresses and canopy cover were used as predictor variables. We also explored the effect of observer effect on number of individuals and species detected in OQ and BQ by plotting the effort (in man-hours) against number individuals and species and testing for correlations. The significance of correlations was tested using Pearson’s product moment correlation. To evaluate the relative efficiency of OQ and BQ in detecting rare species, we plotted the percentage of singletons against sample size [1]. A lower limit of species richness was estimated using Chao1 estimator using the classic formula [35]. For estimating true species richness through extrapolation, we used a second order Jackknife estimate. Comparison of Chao1 and Jackknife estimator was carried out for 47 and 42 samples for both categories of quadrats for lizards and frogs respectively. For a comparison of diversity estimates from OQ and BQ, we used Fisher’s α measure estimated using equal number of randomly selected set of individuals for both OQ and BQ (757 lizards and 188 frogs each). The above analyses were performed using the software EstimateS Ver. 9.1.0 [36], using sampling with replacement and 10,000 iterations. Quadrats from three small islands, where no frogs were detected in any of the sampling strategies (VES or quadrats), were excluded from the calculation of density of frogs. We used Wilcoxon–Mann–Whitney test to assess the significance of difference in estimated densities between OQ and BQ, as well as the difference in effort (measured as man-hours/quadrat).

In order to examine the effect of sampling error on relative abundance of species, we calculated the percentage difference in relative abundance of a species, between OQ and BQ. This was plotted against the rank of species in the community obtained from BQ. Positive values indicated that the species relative abundance was represented more in OQ than in BQ, and negative values indicated the opposite. For exploring bias, we considered estimates from BQ to be very near to the true value [13, 19, 26], so that bias was calculated as a ratio of estimate from OQ to estimate from BQ: a value of 1 indicated an unbiased estimate, while a value less than 1 indicated estimates biased low for OQ. The relative precision of sampling methods was estimated as a ratio of population variance, ${{\hat{\sigma }_{OQ}^{2} } \mathord{\left/ {\vphantom {{\hat{\sigma }_{OQ}^{2} } {\hat{\sigma }_{BQ}^{2} }}} \right. \kern-0pt} {\hat{\sigma }_{BQ}^{2} }}$ after adjusting for the difference in sample sizes. For density estimates, the population variance of BQ was adjusted using the formula $\hat{\sigma }_{BQ}^{2} = \left( {1 - \frac{{n_{OQ} }}{N}} \right)\left( {{{\hat{\sigma }^{{^{ 2} }} } \mathord{\left/ {\vphantom {{\hat{\sigma }^{{^{ 2} }} } {n_{OQ} }}} \right. \kern-0pt} {n_{OQ} }}} \right)$ where, N = total number of potential quadrats, $\hat{\sigma }^{{^{2} }}$ = initial estimate of population variance from BQ, $\hat{\sigma }_{OQ}^{2}$ = population variance estimated from OQ, $\hat{\sigma }_{BQ}^{2}$ = sample-size adjusted population variance estimated from BQ, and $n_{OQ}$ = number of OQs. N was calculated from the cumulative area of all islands sampled. A value of 1 indicated no difference in precision. Values greater than 1 indicated lower relative precision of estimates from OQ than in BQ. For comparing bias and precision of species richness, second order Jackknife estimate was used. These analyses were performed using R [37]. Three arboreal species, namely *Gekko verreauxi*, *Hemidactylus platyurus* and *Pseudocalotes andamanensis*, and snakes were occasionally recorded in quadrats, and were not included in these analyses.

### Availability of supporting data

All the supporting data are included as additional files of the manuscript: Harikrishnan S, Karthikeyan V. Data from: The Devil is in the Detail: Estimating species richness, density, and relative abundance of tropical island herpetofauna. Dryad Digital Repository. doi:10.5061/dryad.88v79.

## Additional files

**Additional files 1:** Abundance and species richness data of frogs in the Andaman Islands collected by using open quadrats and bounded quadrats.
**Additional files 2:** Abundance and species richness data of lizards in the Andaman Islands collected by using open quadrats and bounded quadrats.
**Additional files 3:** File containing details of abbreviations used and the names and areas of islands sampled.

## Abbreviations

BQ: bounded quadrat
OQ: open quadrat
